# Protective effect of calretinin on testicular Leydig cells via the inhibition of apoptosis

**DOI:** 10.18632/aging.101226

**Published:** 2017-04-24

**Authors:** Wendan Xu, Qian Zhu, Bei Zhang, Shan Liu, Xiaonan Dai, Chao Gao, Li Gao, Yugui Cui

**Affiliations:** ^1^ State Key Laboratory of Reproductive Medicine, Clinical Center of Reproductive Medicine, First Affiliated Hospital, Nanjing Medical University, Nanjing 210029, China; ^2^ Center of Reproductive Medicine, Bethune International Peace Hospital, Hebei Shijiazhuang, China; ^3^ Nanjing Maternal and Child Care Service Center, Nanjing Medical University, Nanjing 210005, China

**Keywords:** Leydig cell, calretinin, proliferation, apoptosis, male late-onset hypogonadism

## Abstract

The core mechanism of Late-onset hypogonadism (LOH) is the deficiency of androgen due to the functional and quantitative decline of testicular Leydig cells. Here we explored the protective effect of calretinin, a Ca^2+^-binding protein, on Leydig cells. We found in MLTC-1 cells transfected with LV-calb2, the cell viability and optical density (OD) were higher (*p*<0.05), cells in the S phase of the cell cycle were increased (*p*<0.01) and p-ERK1/2 and p-AKT levels were significantly higher (*p*<0.01 and *p*<0.05), while in R2C cells transfected with LV-siRNA-calb2, all of the results mentioned above were adverse (*p*<0.05). The cell apoptotic index after calretinin over-expressed was significantly lower (*p*<0.001), while the expression levels of mitochondria-related apoptotic factors such as cleaved caspase-9 and cytochrome C (cyto C) were lower and ratio of Bcl2/Bax was higher (*p<*0.05). After calretinin down-regulated, the apoptotic index was higher (*p<*0.05), while the expression levels of mitochondria-related apoptotic factors were higher and the ratio of Bcl2/Bax was lower (*p<*0.05). Therefore, calretinin increases Leydig cell viability and proliferation, possibly via ERK1/2 and AKT pathways, and suppresses apoptosis possibly via the mitochondria-related apoptotic pathway, which could be beneficial in understanding the pathophysiology of LOH and could lead to the study of new treatments.

## INTRODUCTION

Male late-onset hypogonadism (LOH), defined as a clinical and biochemical syndrome characterized by poor morning erection, erectile dysfunction, low sexual desire, sleep disturbances and changes in mood [1, 2], occurs in 30% of 50- to 59-year-old males and 13% of 40- to 49-year-old males [3]. Many other symptoms are also related to LOH, including insomnia, fatigue, muscle mass loss, fat mass increase, bone mineral density decrease and osteoporosis, depression, and forgetfulness [4, 5]. LOH is even considered to be related to substantially higher risks of all-cause and cardiovascular mortality in aging males [6]. It has been demonstrated that the core pathogenesis of LOH is the absolute or relative deficiency of androgen due to the functional and quantitative decline of testicular Leydig cells [7]. There is evidence that disruption of the redox balance within testes and Leydig cells is involved in LOH pathogenesis [8]. However, the mechanism and pathophysiology of LOH are complicated and are not entirely known.

Testosterone replacement treatment (TRT) has been widely used to relieve the main symptoms and to improve the quality of life in aging men with LOH [9-11]. However, some side effects of TRT cannot be ignored, such as erythropoiesis, intrahepatic cholestasis, sleep apnea and liver failure [12]. In addition, TRT is not recommended in men with breast cancer, prostate cancer, or lower urinary tract symptoms due to an enlarged prostate [13]. Because excess exogenous testosterone can suppress the hypothalamic-pituitary-gonadal axis (HPG axis) and spermatogenesis, which lead to male infertility, TRT is also not recommended in males who desire to maintain fertility [14]. Based on the functional improvement and quantitative protection of Leydig cells, a better alternative that increases endogenous testosterone and includes the benefits of TRT without the adverse effects of TRT would be ideal.

Calretinin, also called calcium retinal protein 2 (calb2), is a hexa-EF-hand Ca^2+^ binding protein [15]. The main role of calretinin is as a buffer of intracellular Ca^2+^ to prevent Ca^2+^ overload [16], though it can also function as a Ca^2+^ receptor [17]. The multiple functions of calretinin are mostly dependent on cell type. Previous studies showed that calretinin is mainly expressed in nerve cells and is involved in neuroprotection [18]. Calretinin expressed in granule cells of the cerebellum was also suggested to contribute to information coding and storage [17]. Calretinin was recently found to be expressed in steroidogenic cells, such as adrenal cells and Leydig cells [19]. We previously reported that calretinin was expressed in the cytoplasm of Leydig cells of human, rat and mouse testes [20, 21]. Our previous study also showed that calretinin up-regulated steroidogenesis in Leydig cells via up-regulating steroidogenic enzymes and the PLC-Ca^2+^-PKC pathway. Recently, calretinin expression was detected in many tumors, e.g., mesothelial cells, colon cancer cells, seminomas, Leydig cell tumors and Sertoli cell tumors [22-24].

Calretinin participates in the regulation of the cell viability and proliferation. It was found that the cell activity and proliferation were decreased by calretinin in the mesothelioma cell, and that the G1 phase was retarded while the cell apoptosis was increased via the caspase 9-related pathway [22]. Expression of calretinin was also found in the most of small cell lung cancer cells (SCLS). When the calretinin gene was knocked out in SCLC, the cell apoptosis was significantly increased, suggesting that calretinin inhibited cell apoptosis in SCLC [25]. In addition, studies based on a human colon cancer cell line (WiDr) suggested that the increased expression of calretinin in WiDr cells significantly increased cell proliferation, and that the down-regulated expression of calretinin significantly restrained the cell cycle [23]. It is an interesting research topic whether calretinin play a protective role in the regulation of proliferation and apoptosis of Leydig cells.

The current study was designed to explore the protective effect of calretinin on Leydig cells and to elucidate a possible intracellular mechanism for the protective effect. The LV-calb2 and LV-siRNA-calb2 were successfully constructed in our laboratory. Two Leydig cell lines, R2C and MLTC-1, were used as the *in vitro* models [26]. We found that calretinin played multiple protective roles in Leydig cells via assorted signaling pathways. These results contribute to our understanding of the pathophysiology of LOH and allow us to explore new treatments in the future.

## RESULTS

### Effects of calretinin on Leydig cell viability and proliferation

**Figure 1:**
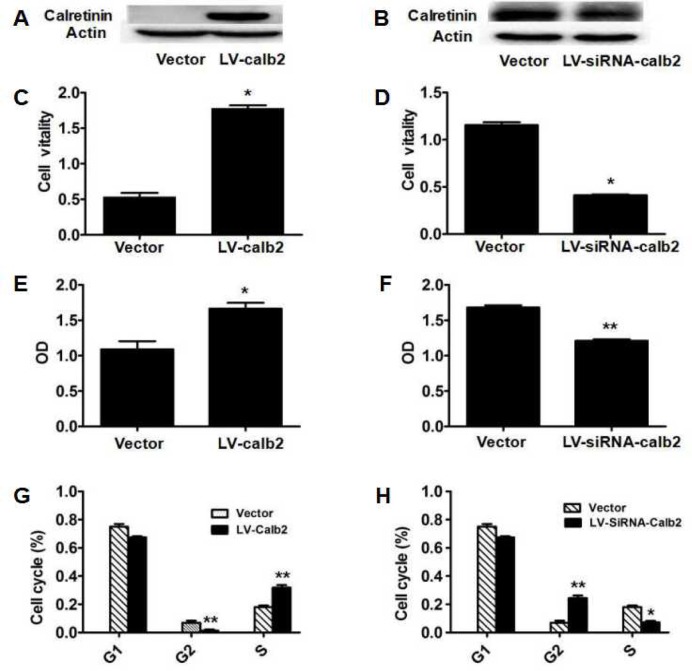
Effect of calretinin on Leydig cell proliferation (**A**) Calretinin expression in MLTC-1 cells transfected with LV-calb2. The vector was used as the negative control. (**B**) Calretinin expression in R2C cells transfected with LV-siRNA-calb2 or vector (as control). (**C**-**H**) After MLTC-1 and R2C cells were transfected with LV-calb2, LV-siRNA-calb2 and vector (as negative control), cell viability was detected using CCK8 kits, cell proliferation was tested with BrdU proliferation assay kits and cell cycle position was analyzed by flow cytometry. (**C**) The viability of MLTC-1 cells with up-regulated calretinin was significantly higher when compared with the control group. (**D**) The viability of R2C cells with down-regulated calretinin was significantly lower. (**E**) The OD of MLTC-1 cells with up-regulated calretinin was significantly higher compared with the control group. (**F**) The OD of R2C cells with down-regulated calretinin was significantly decreased. (**G**) When calretinin was up-regulated in MLTC-1 cells, the number of cells in the G2 phase significantly decreased while the number of S phase cells significantly increased. (**H**) When calretinin was down-regulated in the R2C cells, the number of cells in the G2 phase significantly increased while the number of S phase cells significantly decreased. * *p*<0.05; **: *p*<0.01.

To observe the potential effects of calretinin on testicular Leydig cells, the *in vitro* cultured MLTC-1 cells were transfected with LV-calb2, and the R2C cells were transfected with LV-siRNA-calb2 for 96 h. Calretinin expression in the MLTC-1 cells transfected with LV-calb2 was significantly increased, while calretinin expression in the R2C cells transfected with LV-siRNA-calb2 was significantly decreased (Figure 1A and B). When compared with the respective control groups, the viability in the MLTC-1 cells with over-expressed calretinin was significantly higher (Figure 1C, *p*<0.001), while the viability in the R2C cells with down-regulated calretinin was significantly lower (Figure 1D, *p*<0.05). Meanwhile, the OD was increased in MLTC-1 cells with up-regulated calretinin expression (Figure 1E, *p*<0.05) while the OD was decreased in R2C cells with down-regulated calretinin expression (Figure 1F, *p*<0.05). Interestingly, the number and ratio of cells in the G2 phase of the cell cycle were significantly lower (*p*<0.01) while the number and ratio of cells in the S phase were significantly higher (Figure 1G, *p*<0.01) in the MLTC-1 cells with up-regulated calretinin expression. In the R2C cells with down-regulated calretinin, the number and ratio of cells in the G2 phase of the cell cycle were significantly higher (*p*<0.01) while the ratio of S phase cells was significantly lower (Figure 1H, *p*<0.05).

### Calretinin regulates Leydig cell proliferation partially via the ERK1/2 and AKT pathways

**Figure 2:**
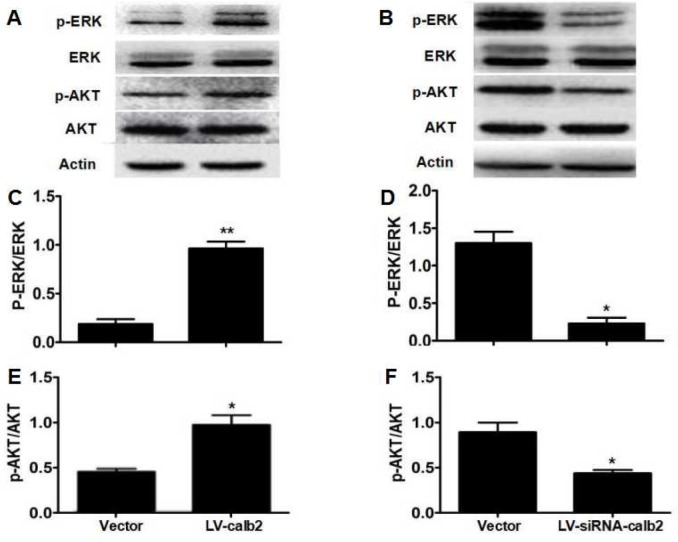
Regulation of calretinin on Leydig cell proliferation partially via the ERK1/2 and AKT pathways (**A**) AKT, p-AKT, ERK1/2 and p-ERK1/2 expression in MLTC-1 cells with over-expressed calretinin. (**B**) AKT, p-AKT, ERK1/2 and p-ERK1/2 expression in R2C cells with down-regulated calretinin. (**C**) The ratio of p-ERK1/2 /total ERK1/2 was significantly higher when calretinin was up-regulated in MLTC-1 cells. (**D**) The ratio of p-ERK1/2/total ERK1/2 was significantly lower when calretinin was down-regulated in R2C cells. (**E**) In MLTC-1 cells with up-regulated calretinin, the ratio of p-AKT/total AKT was significantly higher. (**F**) In R2C cells with down-regulated calretinin, the ratio of p-AKT/total AKT was significantly lower. The vector was used as the negative control(s). * *p*<0.05; **: *p*<0.01.

In the MLTC-1 cells over-expressing calretinin, p-ERK1/2 and p-AKT expression levels were significant-ly up-regulated, and the ratios of p-ERK1/2/total ERK1/2 and p-AKT/total AKT were significantly higher (Figure 2A, C and E, *p*<0.01 and *p*<0.05). In the R2C cells with down-regulated calretinin expression, p-ERK1/2 and p-AKT expression levels were significant-ly down-regulated, and the ratios of p-ERK1/2/total ERK1/2 and p-AKT/total AKT were significantly decreased (Figure 2B, D and F, *p*<0.05).

### Effect of calretinin on Leydig cell apoptosis

**Figure 3:**
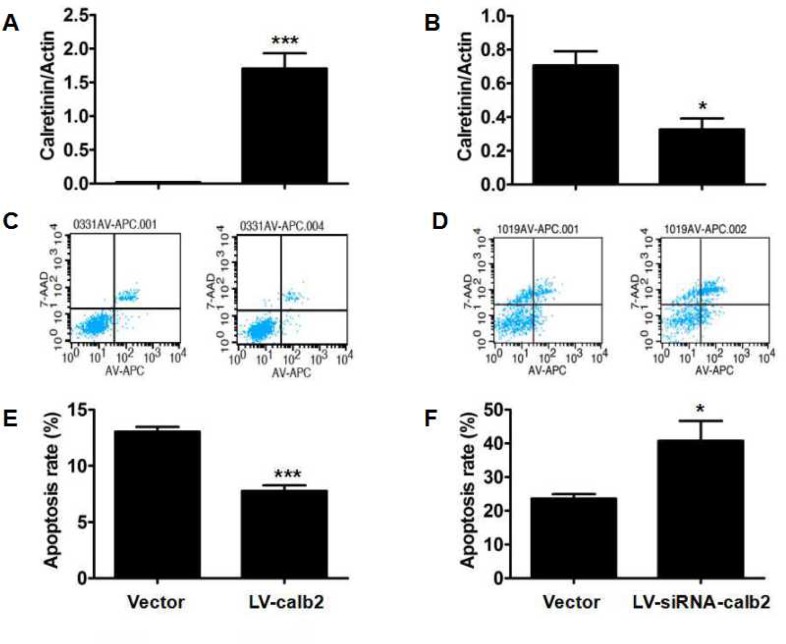
Effect of calretinin on the apoptosis of Leydig cells (**A**) The calretinin expression in the MLTC-1 cells transfected with LV-calb2 was significantly higher (**B**) The calretinin expression in R2C cells transfected with LV-siRNA-calb2 was significantly lower. (**C**) The number of apoptotic cells were significantly decreased in MLTC-1 cells with up-regulated calretinin. (**D**) The apoptotic cells were significantly increased in R2C cells with down-regulated calretinin. (**E**) In MLTC-1 cells transfected with LV-calb2, the apoptotic index was significantly decreased when compared with the control group. (F) In R2C cells transfected with LV-siRNA-calb2, the apoptotic index was significantly higher when compared with the control group. The vector was used as the negative control(s). *: *p*<0.05; ***: *p*<0.001.

Calretinin expression in the MLTC-1 cells transfected with LV-calb2 and the R2C cells transfected with LV-siRNA-calb2 was analyzed by Western blotting (Figure 3A and B). The calretinin expression was significantly up-regulated in the MLTC-1 cells transfected with LV-calb2 (*p*<0.001), while cell apoptosis was decreased and the apoptotic index was significantly lower when compared with the control group (Figure 3C and E, p<0.001). In the R2C cells transfected with LV-siRNA-calb2, the calretinin expression was significantly down-regulated while the apoptotic index was significantly increased (Figure 3D and F, p<0.05).

### Calretinin-mediated inhibition of Leydig cell apoptosis is partially mediated by the mitochondrial-related apoptotic pathway

**Figure 4:**
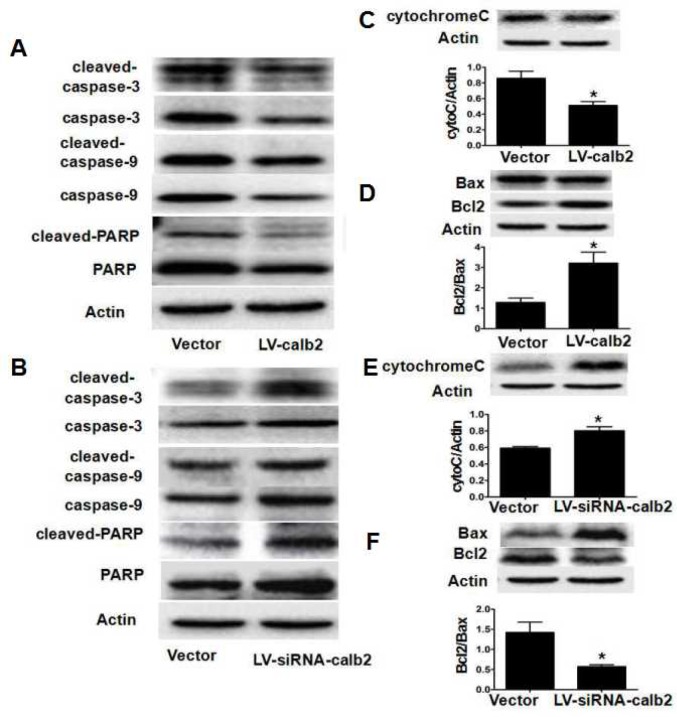
Calretinin inhibits the apoptosis of Leydig cells partially via mitochondrial-related apoptotic pathways After MLTC-1 cells and R2C cells were transfected with LV-calb2, LV-siRNA-calb2 or vector alone, the expression of factors in the mitochondrial-related apoptotic pathways was analyzed by Western blotting. (**A**) In MLTC-1 cells with up-regulated calretinin expression, the caspase-3/9, cleaved caspase-3/9, PARP, and cleaved-PARP expression levels were decreased. (**B**) In R2C cells with down-regulated calretinin expression, the caspase-3/9, cleaved caspase-3/9, PARP, and cleaved-PARP expression levels were increased. (**C**) Cyto C expression was significantly lower in MLTC-1 cells with up-regulated calretinin. (**D**) The Bcl2/Bax ratio was significantly increased in MLTC-1 cells with up-regulated calretinin expression. (**E**) In R2C cells with the down-regulated calretinin, cyto C expression was significantly increased. (**F**) In R2C cells with down-regulated calretinin, the Bcl2/Bax ratio was significantly lower. The vector was used as the negative control(s). *: *p*<0.05.

The expression profiles of factors in the mitochondrial-related apoptotic pathway were measured by Western blotting. In MLTC-1 cells with up-regulated calretinin expression, the caspase-3/9, cleaved caspase-3/9, PARP, and cleaved-PARP expression levels were decreased (Figure 4A), and cyto C expression was significantly lower, while the Bcl2/Bax ratio was significantly higher (Figure 4C and D, *p*<0.05). In R2C cells with down-regulated calretinin expression, the caspase-3/9, cleaved caspase-3/9, PARP, and cleaved- PARP expression levels were higher (Figure 4B), and cyto C expression was significantly higher, while the Bcl2/Bax ratio was significantly lower (Figure 4E and F, *p*<0.05).

## DISCUSSION

In the present study, two testicular Leydig cell lines were used as *in vitro* models to explore the protective effect of calretinin, a Ca^2+^-binding protein, on Leydig cells. In MLTC-1 cells with up-regulated calretinin expression, cell viability and OD were significantly increased while the number of cells in the G2 phase was significantly decreased and the number of cells in the S phase was increased. In the R2C cells with down-regulated calretinin expression, the viability and OD were significantly lower while the number of cells in the G2 phase was significantly higher and the number of cells in the S phase was lower. These results showed that calretinin played a role in enhancing cell viability and in inducing Leydig cell proliferation. Meanwhile, the apoptotic index was significantly decreased by calretinin up-regulation and was significantly increased by calretinin down-regulation, suggesting that calretinin played a role in the inhibition of apoptosis in Leydig cells. Combined with the positive regulation of andro-gen production in our previous study, it can be concluded that calretinin has a protective effect on Leydig cells.

**Figure 5:**
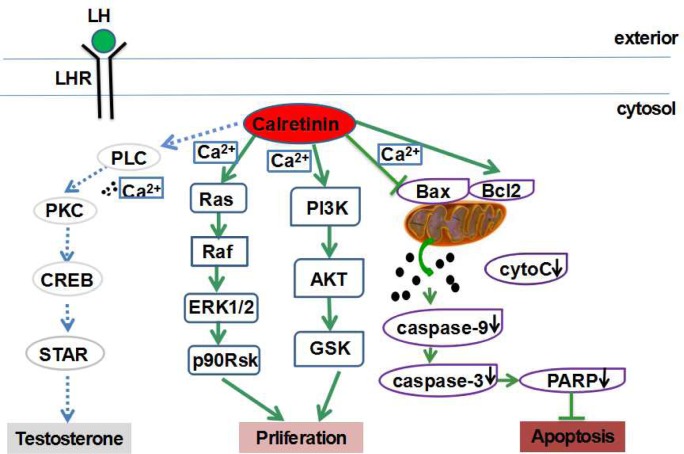
Calretinin plays multiple protective roles in Leydig cells Calretinin increases the cell viability and proliferation of Leydig cells possibly via the activation of the ERK1/2 and AKT pathways, and suppresses cell apoptosis possibly via the inhibition of the mitochondrial-related apoptotic pathway.

Calretinin promotes cell viability and proliferation by comprehensive mechanisms. It was reported that calretinin can protect cells against the cytotoxicity caused by asbestos through the PI3K-AKT pathway [27], and that the PI3K-AKT pathway is one of the classic signaling way to increase cell proliferation [28]. The previous studies also found that the p-ERK1/2 signaling pathway, just like the AKT pathway, was involved in the regulation of the effects of some growth factors and cytokines on cell survival and proliferation [29, 30]. In the present study, p-ERK1/2 and p-AKT expression levels in Leydig cells were significantly up-regulated by calretinin over-expression, and significant-ly down-regulated by calretinin down-regulation in R2C cells, suggesting that the ERK1/2 and PI3K-AKT pathways could be related to the effects of calretinin in Leydig cells (Figure 5). We only observed the expression of those factors in these two pathways after calretinin over-expression or down-regulation in the *in vitro* cultured Leydig cells in this preliminary study. It is necessary to explore the exact signal mechanism of calretinin in regulating cell cycle. Calretinin also played an important role in preventing apoptosis of Leydig cells via the mitochondrial-related apoptotic pathway. There are three types of main apoptotic pathway: the death receptor-mediated apoptotic pathway, the endoplasmic reticulum apoptotic pathway and the mitochondrial-related apoptotic pathway [31-35]. In the present study, we found that apoptosis was inhibited in MLTC-1 cells with up-regulated calretinin expression while the apoptotic index of R2C cells was significantly increased by calretinin down-regulation. Interestingly, the Bcl2/Bax ratio was increased while cyto C, cleaved caspase-3/9 and cleaved-PARP expression levels were significantly decreased in MLTC-1 cells with up-regulated calretinin, which suggested that the mitochondrial-related apoptotic pathway was inhibited (Figure 5). In contrast, the Bcl2/Bax ratio was decreased while cyto C, cleaved caspase-3/9 and cleaved-PARP expression levels were significantly increased in R2C cells with down-regulated calretinin expression.

Calretinin is an important Ca^2+^-binding protein in Leydig cells. Ca^2+^ plays important roles in cell viability and biological affairs and acts as a second messenger, a regulator of ion channels, an activator of protein function, and a promoter of secretion and motion [36]. When the mitochondrial membrane potential is reduced and the mitochondrial membrane permeability is increased by Ca^2+^, cyto C in the mitochondria is released into the cytoplasm, activating caspase-9, and subsequently, caspase-3 and PARP, resulting in cell apoptosis [34]. Moreover, the increased expression of the pro-apoptotic protein Bax, and the reduced expression of the anti-apoptotic protein Bcl2, can decrease the mitochondrial membrane potential and increase the mitochondrial membrane permeability, which promotes cell apoptosis [34]. We propose that calretinin, as a Ca^2+^-binding protein, suppresses the apoptosis of Leydig cells via the inhibition of the mitochondrial-related apoptotic pathway (Figure 5).

The core mechanism of LOH is the deficiency of androgen, especially the active free T, in which three main reasons include the lowered production of testosterone in Leydig cells, the increased level of sex hormone binding globulin (SHBG), and the potential decreased sensitivity of androgen receptor (AR). Many diseases were accompanied with ageing, including chronic diseases such as diabetes, inflammatory arthritis, hypertension, cardiac, hepatic or renal failure, and chronic obstructive lung disease. Besides ageing, these diseases could lead to the functional and quantitative decline of testicular Leydig cells and the increased level of SHBG, although the detailed mechanism is still currently vague [37]. More studies were focused on the pathophysiological mechanism of LOH related to the suppression of androgen production in Leydig cells.

Meanwhile, TRT is the main treatment for LOH patients. TRT improves life quality and prevents diseases related to androgen deficiency. It is necessary to find new biotherapy and adjuvant therapy for LOH to avoid the side effects of TRT. Ali et al. advocated that new therapy should be based on increasing testicular testosterone while maintaining the normal function of LH [37]. Our previous study showed that calretinin enhanced androgen synthesis via the activation of the PLC-Ca^2+^-PKC pathway and by-passed the classic LH/LHR-PKA signaling pathway. In this study, we found that calretinin played multiple protective roles in Leydig cells by increasing cell viability and pro-liferation and inhibiting apoptosis, although the mechanisms underlying these effects are still unclear at present. We propose that calretinin is a new target for the study of steroidogenesis, the pathophysiological mechanisms of LOH, the protection of Leydig cell function and quantity and a new therapy for LOH.

In conclusion, calretinin increases the viability and proliferation of Leydig cells possibly via activating the ERK1/2 and AKT pathways and suppresses cell apoptosis possibly via inhibiting the mitochondrial-related apoptotic pathway. These findings contribute to a better understanding of the pathophysiology of LOH. Calretinin, a Ca^2+^-binding protein, plays multiple protective roles in Leydig cells, suggesting that calretinin is a potential target for further studies to explore new therapies for LOH.

## MATERIALS AND METHODS

### Cells

The mouse Leydig tumor cell line, MLTC-1, was obtained from the Cell Institute of Shanghai (Shanghai, China) and maintained in Roswell Park Institute-1640 medium (RPMI-1640) (Hyclone, Logan, Utah) sup-plemented with 10% fetal bovine serum, 100 U/ml penicillin, and 100 g/ml streptomycin. MLTC-1 cells express calretinin at a low level and were used as a cell model to over-express calretinin as in our previous study.

The rat Leydig tumor cell line, R2C, was purchased from ATCC (Manassas, VA). R2C cells were incubated in DMEM-F12 medium (Gibco, Rockville, MD) supplemented with 15% horse serum and 2.5% fetal bovine serum (FBS) (Gibco, Rockville, MD). R2C cells with high calretinin expression were used as the cell model to down-regulate calretinin in this study. All cells were maintained at 37°C in an atmosphere of 5% CO2.

### Reagents

Cell Counting Kit-8 assay (CCK8) kits, cell cycle assay kits and allophycocyanin (APC) Annexin V/7-aminoactinomycin D (7-AAD flow) cytometric assay kits were purchased from Beijing North Institute of Biological Technology (Beijing, China). The bromodeoxyuridine (BrdU) proliferation assay kits were obtained from Millipore (Billerica, MA). The Bicinchoninic Acid (BCA) kits, pancreatic enzyme, cell lysis, protease inhibitors and phosphorylation protein inhibitors were purchased from Biyuntian (Shanghai, China). The anti-calretinin antibody was obtained from Santa Cruz (Dallas, Texas); the anti-AKT, anti-ERK1/2, anti-p-AKT, anti-p-ERK1/2, anti-caspase-3/9, anti-cleaved caspase-3/9, and anti-PARP antibodies were acquired from Cell Signaling Technology (Beverly, MA); and the anti-cyto C, anti-Bax, and anti-Bcl2 antibodies were obtained from Proteintech Group (Chicago, Illinois). The anti-Actin antibody was purchased from Sigma (Darmstadt, Germany), and anti-rabbit-horseradish peroxidase (HRP)-conjugated and anti-mouse-HRP- conjugated secondary antibodies were obtained from Jackson ImmunoResearch (West Grove, PA). The enhanced chemiluminescence (ECL) kits were purchased from TAKARA (Shiga, Japan).

### Cell transfection

Cultured MLTC-1 cells were transfected with LV-calb2, and R2C cells were transfected with LV-siRNA-calb2, according to the protocol. The multiplicity of infection (MOI) was 100.1×104. The MLTC-1 cells or R2C cells were cultured in 12-well plates. When the cells had grown to 30% confluence, they were transfected with LV-calb2 or LV-siRNA-calb2 and the vector virus (as a control) in the presence of 5 μl/ml of polybrene. At 96 h after transfection, fluorescence was observed with a microscope, and the total cell protein was harvested for Western blot analysis.

### Cell viability assay

Cell viability was assessed using CCK-8 kits according to the manufacturer's instructions. Briefly, MLTC-1 and R2C cells were incubated at a density of 1×10^4 cells/ml in 96-well polystyrene culture plates. After 48 h, 10 μl of CCK8 solution was added to each well. After incubation for 2h at 37°C, the absorbance was determined at 450 nm using an enzyme-linked immuno-sorbent assay (ELISA) plate reader.

### Cell proliferation assay

Cell proliferation was performed using a BrdU cell proliferation kit according to the manufacturer's instructions. Briefly, cells were seeded in 96-well plates at 1×10^4/well, then 20 μl of BrdU labeling solution was added to each well, and the cells were incubated at 37°C for 15 h. Subsequently, 100 μl of anti-BrdU antibody was added to each well and was incubated for 1 hour at room temperature, followed by incubation with peroxidase-conjugated goat anti-mouse IgG for 30 min at room temperature. The substrate used to visualize antibody reactivity was added and incubated for 30 min. Finally, the reaction was measured by determining the OD of the reaction product at a wavelength of 450 nm using an ELISA plate reader.

### Cell cycle analysis

After the MLTC-1 and R2C cells were transfected with LV-calb2 and LV-siRNA-calb2 or the vector virus as a control group, cell cycle analysis was performed according to the manufacturer's protocol. Briefly, MLTC-1 and R2C cells were collected and incubated in 75 % ethanol for 12 h at 4°C and then re-suspended in 500 μl of binding buffer followed by centrifugation at 1,000 x for 5 min at 4°C. The RNA enzyme was then added into the buffer and incubated for 30 min at 37°C. After incubation, 5 μl of propidium iodide (PI) was mixed into each sample and incubated at 20°C for 30 min in the dark. Finally, the cell cycle index was detected using a flow cytometer.

### Annexin V apoptosis assay

MLTC-1 and R2C cells transfected with LV-calb2, LV-siRNA-calb2 or vector virus were harvested and then analyzed according to the manufacturer's instructions. Briefly, the MLTC-1 and R2C cells were mixed with 5 μl of Annexin V-APC and 10 μl of 20 μg/ml PI reagents. The cells were then incubated at room temperature with no light for 20 min. After 400 μl of PBS was added, the samples were subjected to flow cytometry analysis to detect cell apoptosis levels. The apoptotic index was calculated as the sum of the APC-AnnexinV-positive/PI-negative (early apoptosis) and APC-AnnexinV-positive/PI-positive (late apoptosis) cell populations.

### Western blotting

Total cell protein was exacted with radioimmuno-precipitation assay (RIPA) lysis buffer after the MLTC-1 and R2C cells were transfected with LV-calb2 and LV-siRNA-calb2 or vector virus for 96h. The protein concentrations were detected with BCA kits. Approximately 50 μg of protein was separated by SDS-PAGE and transferred to polyvinylidene difluoride (PVDF) membranes. Membranes were blocked with non-fat milk or bovine serum albumin (BSA) at 37°C for 1 h and were then probed with specific anti-calretinin (1:1000), anti-Bax (1:1500), anti-Bcl2 (1:500), anti-cyto C (1:500), anti-AKT (1:1000), anti-ERK1/2 (1:1000), anti-p-AKT (1:1000), anti-p-ERK1/2 (1:1000), anti-caspase-3/9 (1:1000), anti-cleaved caspase-3/9 (1:1000), anti-PARP (1:1000) and anti-Actin (1:10000) antibodies at 4°C overnight. Actin was used as the housekeeping protein in this study. Membranes were washed with TBST 3 times and incubated with secondary antibodies for 1h at 37°C. After incubation, membranes were washed with TBST 3 times again, and the signals were detected using ECL kits. Quantification of bands was measured with the analysis software provided by the imaging system.

### Statistical analysis

All data are expressed as the means ± SD. Every experiment was repeated at least three times from the starting point of cell culture. Student's t-test was used to determine the statistical significance of differences between two groups. All statistical analyses were performed using SPSS 17.0 software. p<0.05 was considered statistically significant.

## Abbreviations

LH: Luteinizing hormone
HPG: hypothalamic-pituitary-gonadal
calb2: calcium retinal protein 2
SCLS: small cell lung cancer cells
WiDr: human colon cancer cell line
FBS: fetal bovine serum
CCK8: Cell Counting Kit-8 assay
Brdu: bromodeoxyuridine
BCA: bicinchoninic acid
EDTA: ethylenediaminetetraacetic acid
APS: ammonium persulfate
SDS: sodium dodecylsulfate
Tris: tris hydroxymethyl aminomethans
LOH: Late-onset Hypogonadism
DMSO: dimethyl sulfoxide
OD: optical density
HRP: horseradish peroxidase
TEMED: tetramethyl ethylenedamine
PVDF: polyvinylidene difluoride
DMEM-F12: Dulbecco's Modified Eagle Media: Nutrient Mixture F-12
APC: Allophycocyanin
BSA: bovine serum albumin
PVDF: polyvinylidene difluoride
RIPA: radioimmunoprecipitation assay
PI: pidium iodide
MOI: multiplicity of infection
ECL: enhanced chemi-luminescence
